# Comparative study of sample storage conditions on gut dysbiosis in peripheral artery disease

**DOI:** 10.21203/rs.3.rs-7483387/v1

**Published:** 2025-09-22

**Authors:** Sarbjeet Niraula, Imadh Khan, Jonathan Jung, Spencer L. Stirewalt, Megan Alagna, James Du, Liqun Xiong, Ayman Elmasri, Erik Wu, Patrick Seed, Stefan J. Green, Karen J. Ho

**Affiliations:** Northwestern University; Southern Illinois University School of Medicine; University of Washington School of Medicine; University of Chicago; Northwestern University; University of Illinois at Chicago; Northwestern University; Southwest Healthcare; Northwestern University; Ann & Robert Lurie Children’s Hospital of Chicago; Rush University; Northwestern University

**Keywords:** Humans, Gastrointestinal Microbiome, Healthy Volunteers, Dysbiosis, Microbiota, Peripheral Artery Disease, Biomarkers

## Abstract

**Background:**

Large-scale gut microbiome studies rely on fecal sample storage prior to batch sample preparation, sequencing, and analysis. Effects of storage methods have largely been studied using samples from healthy participants, where the microbial communities and the metabolic environment are in concordance. In diseased states, dysbiosis is more prone to environmental perturbation, which causes variable shifts in the communities. Cardiovascular diseases are associated with gut dysbiosis, but the effect of storage methods on the qualitative and quantitative aspects of dysbiosis is unknown. Thus, we examined the effects of 3 sample storage conditions on the fecal samples of patients with peripheral artery disease (PAD), a form of cardiovascular disease, and non-PAD controls.

**Methods and Results:**

This is a cross-sectional study of fecal samples collected from adults with PAD and non-PAD controls. All participants (12 non-PAD and 18 PAD) followed the home fecal sample collection protocol. Each sample was immediately frozen (IF), placed in modified Cary-Blair (CB), and stored in an OMNIgene•Gut vial. All samples were subjected to 16S rRNA gene amplicon sequencing of the hypervariable V4 region. A subset of glycerol stocks from IF and CB samples was thawed and cultured to compare revivification. We found significant differences in microbial composition and community structure between non-PAD and PAD groups based on storage conditions. Although we did not see the effect of an interaction term (disease group*storage condition) at the community level, we observed storage condition-specific differential abundance of genera in the PAD compared to the non-PAD group. The high number of differentially variable taxa in the PAD group samples further emphasize the need for standardizing storage conditions. The subset of samples stored in CB had less revivification potential than IF samples under both anaerobic and aerobic processing conditions.

**Conclusions:**

Sample storage conditions and room temperature storage time differentially affect the microbial communities of fecal samples and revivification of glycerol stocks from non-PAD and PAD groups. The effects of storage conditions can bias microbiome-related disease biomarker discovery. Careful consideration should be given to sample storage conditions when analyzing fecal samples from diseased populations and when combining data from cohorts with samples stored in different conditions.

## Background

High-throughput sequencing technologies have enabled the study of the human gut microbiome in unprecedented detail. (1–3) These are based mainly on microbial genomic content extracted from fecal samples due to the non-invasive and low-cost nature of the sample collection process. (4) Fecal sample collection from participants in non-lab settings, followed by sample transport, processing, and long-term storage, can introduce biases due to overgrowth of specific microbes or degradation of genomic contents. (5, 6) While rapid freezing to −80°C has been reported as a best practice, (7–9) this may not be feasible in all studies, including those that utilize home fecal sample collection. Various sample storage media, conditions, and processing methods have been described for preserving fecal samples for microbiome research. (5, 6, 10–20) However, variables such as storage temperature, (21, 22) duration of storage, (23, 24) and type of storage media (5, 6, 10, 12–15, 17–20, 25) influence the integrity of microbial DNA. Prior studies by others have evaluated the effects of sample preservation methods such as Cary-Blair (CB) media, (16) OMNIgene•GUT (10, 15, 17, 20, 24–28), RNAlater, (9, 10, 13, 17, 28) ethanol (6) (Song et al., 2016), glycerol phosphate buffer, (29) N-octylpyridinium bromide, (30) Whatman FTA cards (6) and Zymo DNA/RNA shield (31) and others in fecal microbiome profiles. (6, 9, 25, 32) CB media is an isotonic, non-nutritive, buffered solution that is commonly used for the collection of enteric pathogens. (16, 33) OMNIgene•GUT kit is a commercially available kit that has been found to stabilize microbial DNA for more than 7 days at room temperature (34) and provides reproducible microbiome profiles (10, 15, 17, 20, 24–28) but is costly and preserved samples cannot be used in downstream culture-dependent experiments.

Prior studies on sample collection and preservation methods have mainly utilized fecal samples from healthy individuals. (6, 9, 16, 25) While dysbiosis has been described in multiple cardiovascular diseases such as coronary artery disease, (35–38) hypertension, (39–44) stroke, (45–47) and peripheral artery disease (PAD) (48), the impact of sample preservation methods on the quantitative and qualitative aspects of dysbiosis in these conditions is not well understood. Thus, we performed a comparative cross-sectional analysis of the differential effects of sample storage conditions on samples from individuals with PAD and non-PAD controls. We used home fecal sample collection followed by 3 storage conditions, immediate freezing (FR), modified CB media, and OMNIgene•GUT (GT). We used 16S rRNA gene amplicon sequencing and anaerobic cultivation to investigate storage condition-dependent variations in both the sequencing profiles and revivification. We hypothesized that storage conditions would result in differential characterization of PAD dysbiosis and sample viability following revivification.

## Methods

### Study participants

Adults diagnosed with PAD and adults without PAD who presented to Northwestern Memorial Hospital (Chicago, Illinois, USA) outpatient vascular surgery clinic were screened by the research team. Exclusion criteria included inability to provide informed consent or follow the study protocol, antibiotic use within the prior 3 months, and bowel surgery or bowel preparation within the prior 3 months. After obtaining informed consent, instructions about home fecal sample collection and a collection kit were provided.

Participants were asked to return the samples to the study team within 48 hours of collection. Demographic and clinical information were obtained from the electronic medical record or a health questionnaire. This study was approved by the Northwestern University Institutional Review Board (Protocols #STU00213201 and #STU00216722).

### Fecal sample collection and processing

**Figure 1:**
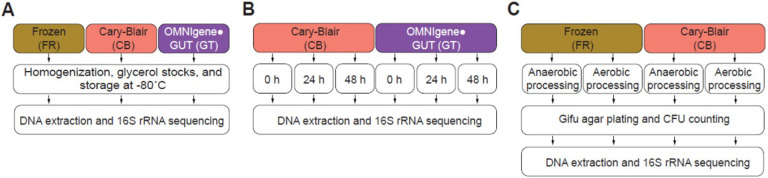
Experimental design. **(A)** Home fecal sample collection was performed and samples were immediately divided into 3 storage conditions. Samples were returned to the lab within 48 hours and stored at −80°C prior to 16S rRNA sequencing. **(B)** A subset of Cary-Blair (CB) and OMNIgene·Gut (GT) samples were each stored at room temperature (RT) for 0, 24, and 48 samples before 16S rRNA sequencing. **(C)**Glycerol stocks from immediately frozen (FR) and CB samples were used for anaerobic cultivation, followed by DNA extraction and 16S rRNA sequencing.

Fecal sample collection kits included a stool collection hat (Fisher Scientific, USA), empty stool transport vial (Meridian Bioscience, USA), a stool transport vial containing modified CB media (Fisher Scientific, USA), and an OMNIgene•Gut collection kit (DNA Genotek, USA). Participants were instructed to aliquot stool into each container immediately after collection. The stool transport vial was then immediately frozen (FR), while the CB and GT samples were kept at ambient temperature. The frozen sample was kept frozen during transport with an insulated bag and ice pack. Upon reaching the lab, samples were homogenized and aliquoted in 1.5 mL screw cap O-ring tubes (Dot Scientific, USA) and immediately placed in the − 80°C freezer (Fig. 1A). The processing was done aerobically on the benchtop as quickly as possible. A subset of samples was processed both aerobically and anaerobically in a separate analysis (see below). The total storage time was defined as the time samples were produced until processing was completed. Similarly, a subset of non-PAD (n = 5) and PAD (n = 5) samples from CB and GT storage media were aliquoted and kept at room temperature and DNA was extracted 0, 24, and 48 hours prior to sequencing (Fig. 1B).

### DNA extraction and library preparation

DNA was extracted using the PowerSoilPro DNA isolation kit (QIAGEN, Germany) followed by a two-stage PCR amplification protocol, as described previously. (49) Briefly, the first stage of PCR amplification was performed using a primer set targeting the microbial 16S rRNA gene V4 hypervariable region. These primers contained “common sequence” linkers CS1 and CS2 (i.e., CS1_515F acactgacgacatggttctacaGTGYCAGCMGCCGCGGTAA and CS2_806R tacggtagcagagacttggtctGGACTACNVGGGTWTCTAAT; linkers are underlined). Amplicons were prepared for Illumina sequencing by a 2nd PCR employing Access Array Barcode Library for Illumina Sequencers (Fluidigm, USA). PCR conditions were described previously. (50) ZymoBIOMICS Microbial Community DNA Standard (Zymo Research, USA) was used as a positive control to evaluate biases in library preparation, sequencing, and sequence processing. Libraries were sequenced on an Illumina MiniSeq (PE 2×154 bp) instrument at the Rush Genomics and Microbiome Core Facility (GMCF; Chicago, USA).

### Bioinformatics analysis

Raw sequences were analyzed using Qiime2 (v2023.2.0) (51) and PhyloSeq (v1.50.0) (52) pipelines. Degenerate primer sequences were trimmed using q2-cutadapt.(53) Trimmed sequences were quality filtered using dada2. (54) Approximately 80% of the total raw sequence counts were specified to train the error model and pseudo pooling was used for denoising. Prevalence-based contaminants were filtered using the decontam package in R. (55) Samples from different batches were preprocessed independently, and amplicon sequence variant (ASV) tables were merged. Batch effect was not corrected as we did not observe significant batch effects from two sequencing runs. Sequences were classified using trained and human stool weighted (56, 57) classifier from the Greengenes2 2022.10 database.(58) Features with ambiguous sequences (poly-A and poly-G tails) were discarded. Only sequences that could be classified to the phylum level or higher were retained, and those annotated as mitochondria and chloroplast were filtered out.

A phylogenetic tree was constructed by fragment insertion using q2-fragment-insertion with SEPP using the Greengenes 13.8 database.(59, 60) All ASVs were inserted into the reference tree. For taxonomic classification, sequences were aligned with the Greengenes2 full length backbone followed by tree-based annotation.(59, 60) Phylogeny-based taxonomies of 16S amplicon sequences has shown better accuracy and high correlation with the shotgun annotation method at species level compared to the conventional Naïve Bayes method.(58, 60) Unaligned ASVs were classified up to the genus level by using q2-classifier classify-sklearn against GreenGenes2 2022.10 that was trained with 515F/806R primers and weighted for the human stool environment. Sequences were rarefied to minimum sequencing depth per samples (i.e., 14,299 to 28,142 reads) for alpha and beta diversity analysis and all samples were retained.

### Revivification and sample viability testing

Only FR and CB samples were used for viability testing, since OMNIgene•Gut technology does not preserve cell viability. A subset of 12 samples (8 non-PAD and 4 PAD) from each of the original FR and CB groups was processed simultaneously in both anaerobic (i.e., in an anaerobic chamber) and aerobic (on the benchtop) environments into 10% glycerol stocks (GS) and frozen at −80°C (Fig. 1C). GS were thawed in a 37°C water bath, homogenized, and serially diluted in phosphate-buffered saline (PBS). Diluted samples were plated on Gifu Anaerobic Medium (GAM) agar (HiMEDIA, USA) and incubated at 37°C in an anaerobic chamber. Colony forming units (CFU) were counted at 24-hour intervals for 96 hours. At the 96-hour time point, colonies were mixed with 10% glycerol in PBS and frozen at −80°C. DNA was extracted from both glycerol stocks and the cultured colony mixture, and 16S rRNA gene amplicon sequencing was performed as described above.

### Statistical analysis

The final ASV table, taxonomy, and reference tree were imported to PhyloSeq using Qiime2R (v0.99.6). (61) Alpha diversity metrics such as observed richness, Shannon index, Simpson index, and Pielou Evenness were calculated using microbiome (v1.28.0). (62) Beta diversity was calculated using unweighted- and weighted UniFrac distance matrices. Beta diversity was compared using permutational multivariate analysis of variance (PERMANOVA) using the adonis2 function in vegan (v2.6–10). Differential abundance and differential variability analysis were performed using corncob (v0.4.1). (63) Species found in at least 3 samples in each comparison group were included for differential abundance analysis using the likelihood ratio test (LRT). Taxa were considered differentially abundant and differentially variable if the model adjusted (false discovery rate, FDR) p ≤ 0.05 and taxa adjusted (FDR across all taxa) p ≤ 0.1. Since the sample size was small, adjustment for covariates (age, sex, race, body mass index, smoking, storage time, and sequencing batch) was performed by reducing dimensionality using FAMD in FactoMineR (v2.11). (64) The first four principal components were used as covariates. For the time series analysis, we used the package MicrobiomeStat (v1.2.1). (65) Interaction effects between study group, storage condition, and storage time were calculated using the zero-inflated beta-binomial model in glmmTMB (v1.1.10), (66) followed by assessment of goodness-of-fit of each model using DHARMa (v0.4.7). (67) In the glmmTMB model, subjects were selected as a random intercept, and the library size was used as an offset term. P values were FDR-adjusted across all taxa. Log fold change (lfc) was assessed using the linear discriminant analysis (LinDA) function. Statistical analysis was performed in R (v4.4.2) and GraphPad Prism v10.

## Results

### Study participants and fecal samples

There were 35 participants, 21 with PAD and 14 non-PAD controls. All participants followed the sample collection protocol, aliquoted the samples into 3 different storage conditions, and returned all 3 samples within the specified timeframe. Demographic and clinical characteristics are shown in Table 1.

### Effects of storage condition on microbiome diversity

**Figure 2:**
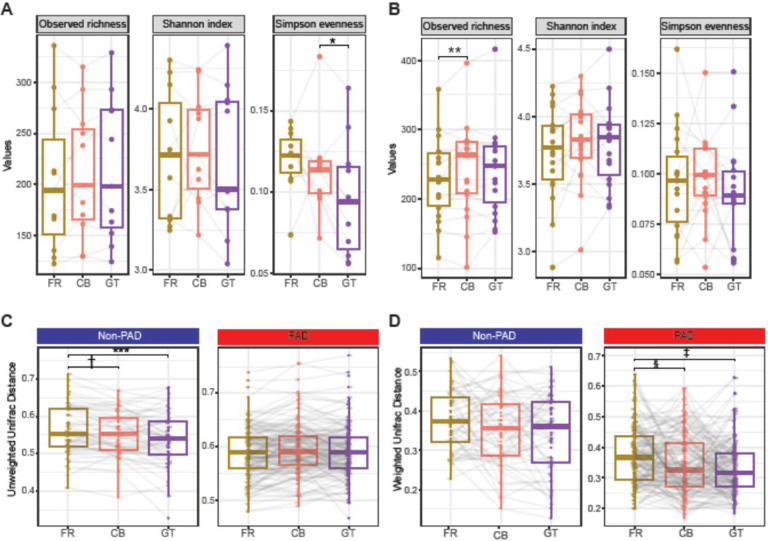
Comparison of alpha and beta diversity between the 3 storage conditions. Differences in alpha diversity between the 3 storage conditions in the non-PAD (*, p_adj_ = 0.014) **(A)** and PAD (**, p_adj_ = 0.026) **(B)** groups. Differences in unweighted UniFrac (†, p_adj_ = 0.009; ***, p_adj_ = 0.002) **(C)** and weighted UniFrac (§, p_adj_ = 0.005; ‡, p_adj_ = 7.08e^−06^) **(D)** distances in the 3 storage conditions in the non-PAD and PAD groups. P values are Benjamini-Hochberg adjusted.

The effects of storage conditions on alpha and beta diversity were analyzed separately for the PAD and non-PAD groups. The observed richness of ASVs was similar in all 3 storage conditions in the non-PAD group (Fig. 2A), whereas CB samples had higher observed richness than the FR (p = 0.026) samples in the PAD group (Fig. 2B). Shannon index was similar in all 3 storage conditions in both PAD and non-PAD groups. Simpson evenness was similar between the FR and CB samples and decreased in the GT samples (p = 0.014) in the non-PAD group (Fig. 2A) and was similar in all 3 conditions in the PAD group (Fig. 2B).

We measured community composition using unweighted UniFrac distance and community structure using weighted UniFrac distance matrices in non-PAD and PAD samples in all 3 storage methods. The average median value of the unweighted UniFrac distances measured within the FR samples in the non-PAD group was significantly higher than those observed in CB (p_adj_ = 0.009) and GT (p_adj_ = 0.002) samples (Fig. 2C). The average median value of the weighted UniFrac distances measured within the FR samples in the PAD group was significantly higher than those observed in CB (p_adj_ = 0.005) and GT samples (p_adj_ = 7.08E^− 06^) (Fig. 2D). This suggests that the selection of storage condition affects the similarity between samples within the experimental group based on the choice of distance matrices. However, the overall community composition (unweighted UniFrac distance) of non-PAD and PAD groups was similar in the 3 storage conditions (**Supplemental Fig. 1A and 1B**), whereas the structure (weighted UniFrac distance) differed significantly (non-PAD group, **Supplemental Fig. 1C** and PAD group, **Supplemental Fig. 1D**). The pairwise weighted UniFrac distance was highest between FR and GT samples (non-PAD F = 14.22, p = 0.0003; PAD F = 14.70, p = 0.0003) compared to FR and CB (non-PAD F = 3, p = 0.018; PAD F = 5.62, p = 0.001) and CB and GT (non-PAD F = 5.2, p = 0.003; PAD F = 2.58, p = 0.03).

The Mantel correlation coefficients of distances between samples in the 3 storage conditions were higher (Spearman’s rho = 0.72 to 0.87, p = 0.0001) using the unweighted UniFrac distances than the weighted UniFrac distances (Spearman’s rho = 0.27 to 0.54, p = 0.0001) (**Supplemental Table 1**). The correlation coefficients of unweighted UniFrac distances for all storage conditions were significantly higher (p = 0.0019) in the non-PAD group than in the PAD group (**Supplemental Table 1**).

### Effects of storage condition on PAD dysbiosis

**Figure 3:**
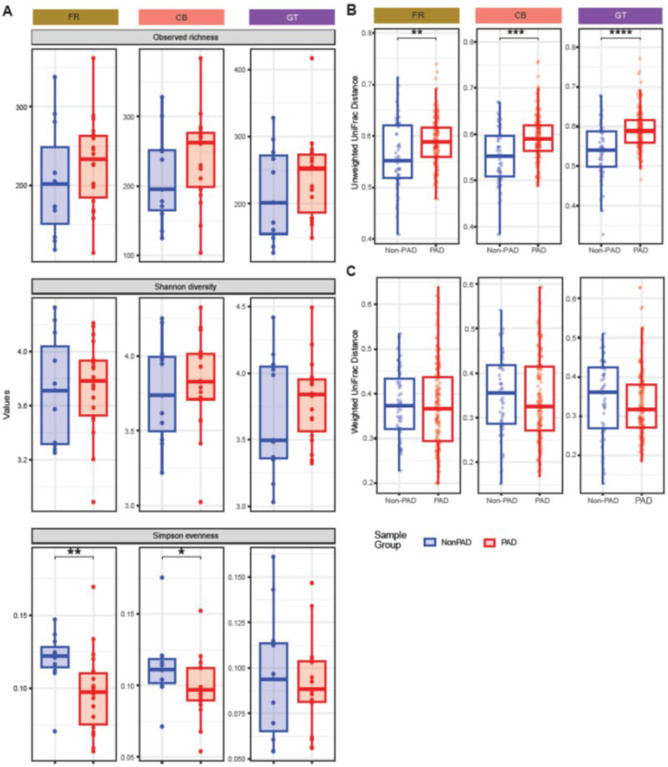
Comparison of alpha and beta diversity between the non-PAD and PAD groups. Differences in alpha diversity between the non-PAD and PAD groups **(A)** (*, p = 0.044; **, p = 0.006). Differences in unweighted UniFrac **(B)** (**, p = 0.008; ***, p = 8.3e^−06^; ****, p = 9e^−07^) and weighted UniFrac **(C)** distances between the non-PAD and PAD groups.

There were no differences in observed richness or Shannon diversity between the non-PAD and PAD groups in any storage condition (Fig. 3A). The PAD group had significantly lower Simpson evenness than the non-PAD group in only the FR and CB samples (p = 0.006 and p = 0.044, respectively) (Fig. 3A). The average median unweighted UniFrac distance within the non-PAD group was significantly less than the PAD group, suggesting that the communities are more diverse among patients with PAD (Fig. 3B), whereas the median weighted UniFrac distances within these groups were similar in all 3 storage conditions (Fig. 3C). The overall variability, or the dispersion of unweighted UniFrac distances from the centroid, was similar between non-PAD and PAD groups in FR samples (p = 0.139), and dissimilar between CB (p = 0.025) and GT (p = 0.012) samples (**Supplemental Fig. 2A**). The dispersion of weighted UniFrac distances between non-PAD and PAD groups was similar in all 3 storage conditions (**Supplemental Fig. 2B**).

Although there were no significant differences in beta diversity between the non-PAD and PAD groups using unweighted and weighted UniFrac distance matrices (data not shown), the unweighted UniFrac distance between non-PAD and PAD groups was least in GT samples compared to FR (p = 0.043) and CB (p = 0.0001) samples (**Supplemental Fig. 2C**). On the other hand, the weighted UniFrac distance between PAD and non-PAD groups was highest in FR compared to CB (p = 0.01) and GT (p = 0.03) samples (**Supplemental Fig. 2D**), suggesting that differences in microbial community between the non-PAD and PAD groups were the most distinct in the FR storage condition.

### Differential abundance and differential variability of species-level taxonomic features

**Figure 4:**
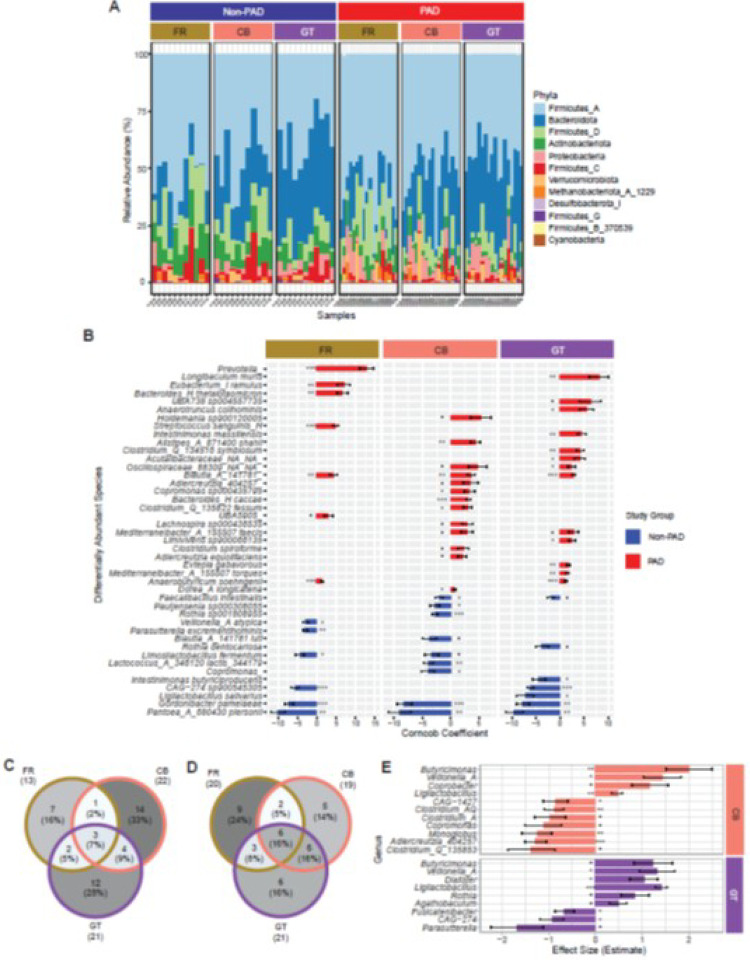
Comparison of taxonomy in clinical groups and storage conditions. Relative abundance of phyla by clinical group and storage condition **(A)**. Differentially abundant species between the non-PAD and PAD groups in each storage condition **(B)**. Venn diagram of common (shared) differentially abundant species **(C)** and differentially variable species **(D)** between the 3 storage conditions. Comparisons are made between the non-PAD and PAD groups. **(E)** Genera associated with the interaction term of clinical group and storage condition.

Relative abundance of taxa at the phylum level showed high abundance of Firmicutes_A in both non-PAD and PAD groups, followed by Bacteroidota, Firmicutes_D, Actinobacteriota, and Proteobacteria in all storage conditions (Fig. 4A). However, the relative abundance of Bacteroidota was lower in FR samples compared to CB and GT in both non-PAD and PAD groups (Fig. 4A). A total of 13, 22, and 21 differentially abundant species (p_adj_ < 0.1) between non-PAD and PAD groups were identified in the FR, CB and GT storage conditions, respectively (Fig. 4B), after adjusting for the first 4 principal components that represent 69.98% of the combined effect of the covariates age, sex, race, smoking, BMI, storage time and sequencing batch (**Supplemental Fig. 3A**). Among them, 3 (7%) were commonly identified by all 3 storage media, and 16%, 14% and 12% were unique to FR, CB and GT samples, respectively (Fig. 4C). Species such as *Blautia_A_*141781 were more differentially abundant in PAD samples whereas *Gordonibacter pamelaeae* and *Pantoea_A_680430 piersonii* were less abundant in all 3 storage media (Fig. 4B). On the other hand, *G. pamelaeae* is differentially variable in the non-PAD group and *Blautia_A_141781* is differentially variable in the PAD group (**Supplemental Fig. 3B**). Similarly, we identified 20, 19 and 21 differentially variable species between non-PAD and PAD groups in FR, CB and GT samples, respectively (Fig. 4D, **Supplemental Fig. 3B**). Among the differentially variable species, 16% were commonly identified by all 3 storage media (Fig. 4D). The number of differentially variable species were higher in the PAD group compared to the non-PAD group in all 3 storage media, suggesting that dysbiosis is affected by (1) inter-individual heterogeneity in how PAD is associated with the microbiome and by (2) unmeasured heterogeneity in the PAD phenotype in our cohort.

The odds of having higher abundance of genera such as *Butyricimonas* (CB Coeff. = 2.01, *p* = 0.002; GT Coeff. = 1.23, *p* = 0.07), *Veilonella_A* (CB Coeff. = 1.44, *p* = 0.01; GT Coeff. = 1.32, *p* = 0.02) and *Ligilactobacillus* (CB Coeff. = 0.46, *p* = 0.003; GT Coeff. = 1.4, *p* = 4.62e^− 28^) are higher in CB and GT storage conditions compared to FR in the PAD group (Fig. 4E). On the other hand, genera such as *Copromonas* (Coeff. = −1.11, *p* = 0.07) and *Clostridium* in CB samples and *Fusicatenibacterci* (Coeff. = −0.66, *p* = 0.036) and *Parasutterella* (Coeff. = −1.67, *p* = 0.07) in GT samples were found to be less abundant in PAD (Fig. 4E). These results suggest that there are storage condition-specific biases in the identification of differentially abundant taxa.

### Effects of storage condition on revivification of microbial communities

**Figure 5:**
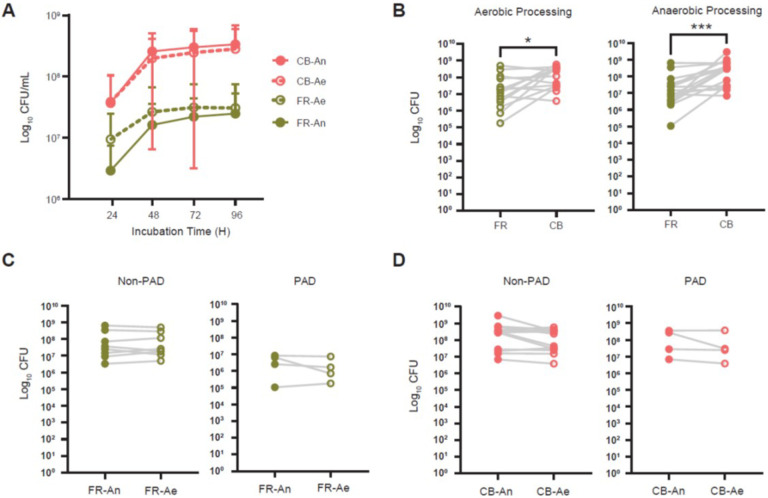
Revivification of microbiota in FR and CB samples following aerobic and anaerobic processing. **(A)** Serial colony forming units (CFUs) obtained on Gifu agar media. **(B)** Differences in CFUs after aerobic and aerobic processing (*, p = 0.04; ***, p = 0.0004). **(C)**Comparison of CFUs after anaerobic and aerobic processing followed by anaerobic cultivation.

We next compared the revivification of FR and CB glycerol stocks (GS) that were processed aerobically or anaerobically and then cultured anaerobically on GAM agar. CFUs obtained every 24 hours was similar between FR and CB with no significant differences based on the processing environment (Fig. 5A). FR and CB samples both reached a plateau in CFU after 48 hours. CB samples had higher CFUs at 48 hours compared to FR samples after both anaerobic (p = 0.0004) and aerobic (p = 0.035) processing (Fig. 5A and 5B). Within the non-PAD and PAD study groups, there were no differences in CFU between anaerobically and aerobically processed samples in either FR or CB storage conditions (Fig. 5C and 5D).

**Figure 6:**
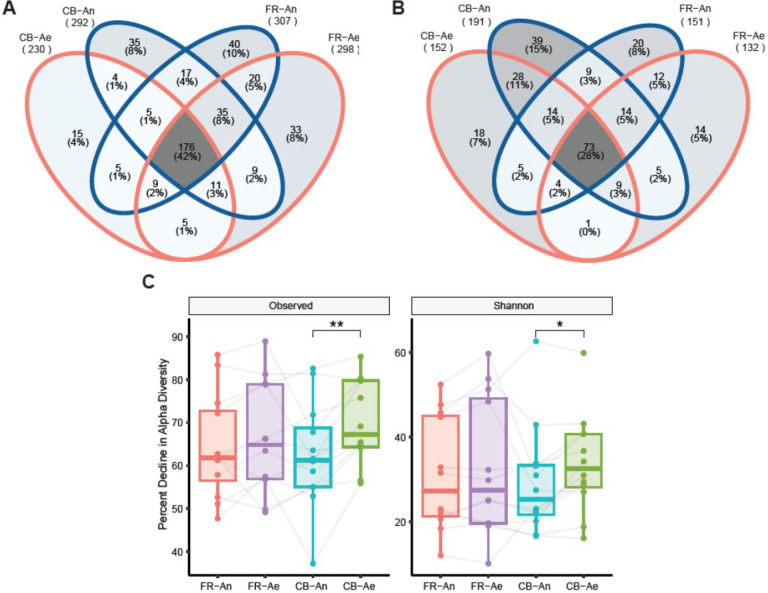
16S rRNA gene amplicon sequencing of colonies and glycerol stocks (GS). Venn diagram of shared amplicon sequence variants (ASVs) obtained from non-PAD **(A)**and PAD **(B)** samples. **(C)** Percent decline in alpha diversity between colonies and glycerol stocks (*, p = 0.021; **, p = 0.0015).

Based on 16S rRNA gene amplicon sequencing of the GS and the colonies collected after the 96-hour time point, we obtained a total of 1203 and 470 ASVs in the GS and colonies, respectively. Among non-PAD samples, a higher percentage of ASVs were identified as common to the 4 storage and processing conditions (FR aerobic, FR anaerobic, CB aerobic, and CB anaerobic) compared to PAD samples (42% non-PAD vs. 28% PAD) (Fig. 6A and 6B), suggesting that the effect of storage condition and processing method is more pronounced in disease-associated gut microbiota compared to normal gut microbiota. There was a significant decline in observed richness and Shannon diversity in GAM agar cultures from aerobically processed CB GS compared to anaerobically processed CB GS (p < 0.01; Fig. 6C and 6D), suggesting that the anaerobic microbes diluted in reagents are more prone to oxidative stress in short term exposure to air.

We performed Mantel correlation of the unweighted distance matrices of communities from GS and plated colonies from all 4 storage conditions and processing methods (FR aerobic, FR anaerobic, CB aerobic, and CB anaerobic). Correlations between the structure (unweighted UniFrac distance) of cultured communities on GAM agar and their respective GS were highest in anaerobically processed FR samples (FR-An) (Spearman’s rho = 0.7, p = 1e^− 04^) (**Supplemental Fig. 4A**), followed by anaerobically processed CB samples (CB-An) (Spearman’s rho = 0.61, p = 3e^− 04^) (**Supplemental Fig. 4C**), aerobically processed FR samples (FR-Ae) (Spearman’s rho = 0.4, p = 0.03) (**Supplemental Fig. 4B**) and aerobically processed CB samples (CB-Ae) (Spearman’s rho = 0.38, p = 1e^− 04^) (**Supplemental Fig. 4D**), demonstrating that freezing samples at home followed by anaerobic processing is a better alternative to the use of reagent-based storage media if downstream revivification of samples is anticipated.

### Effects of storage condition on microbial communities stored at room temperature

**Figure 7:**
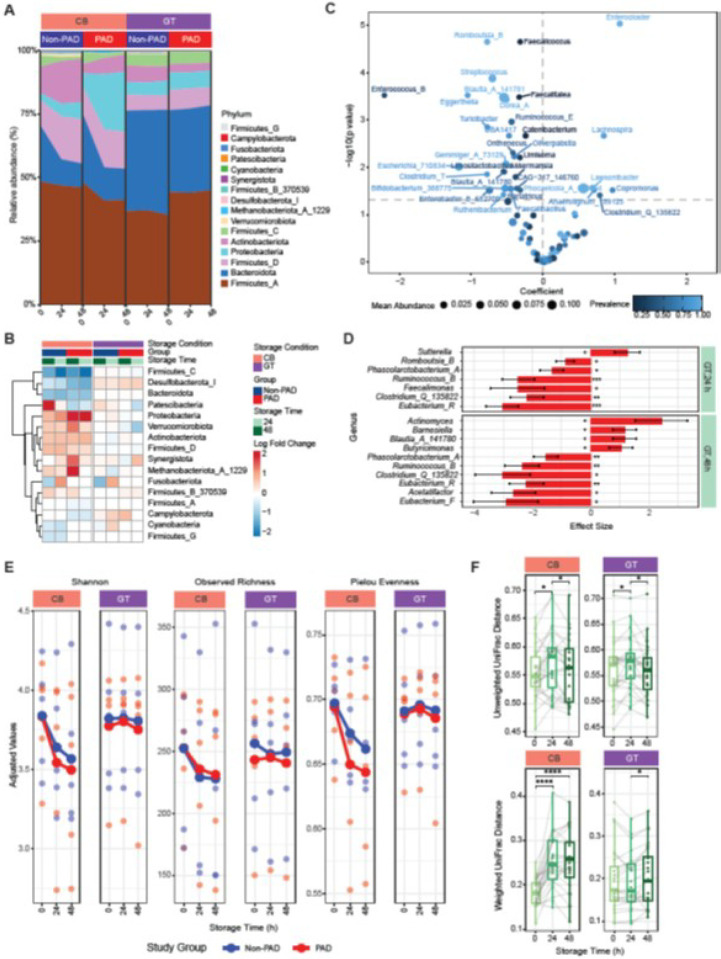
Room temperature (RT) storage introduces differences in microbial community in CB and GT samples. **(A)** Composition of phyla in CB and GT samples after 0, 24, and 48 hours of RT storage. **(B)** Log-fold change in phyla after 24 and 48 hours of RT storage compared to 0 hour (baseline) samples. **(C)** Differential abundance of genera in CB and GT samples. **(D)** Interaction effects of storage condition and storage time on differential abundance in the PAD group. *, p < 0.05. **, p < 0.01, ***, p < 0.001. **(E)** Changes in alpha diversity in CB and GT samples over time at RT. **(F)** Differences in unweighted and weighted UniFrac distances over time at RT. Unweighted UniFrac, CB, 0–24 hour, *, p = 0.016; Unweighted UniFrac, CB, 24–48 hour, *, p = 0.016; Unweighted UniFrac, GT, 0–24 hour, *, p = 0.028; Unweighted UniFrac, GT, 24–48 hour, *, p = 0.02; Weighted UniFrac, CB, 0–24 hour, ****, p = 1.25e^−06^; Weighted UniFrac, CB, 0–48 hour, ****, p = 1.52e^−05^; Weighted UniFrac, GT, 24–48 hour, *, p = 0.026.

Using a subset of CB and GT samples from non-PAD and PAD groups, we analyzed the effect of room temperature (RT) storage on the relative change in bacterial communities. We observed uniform relative abundance over time in both storage conditions and groups (Fig. 7A). Log fold increase of Proteobacteria after 24 and 48 hours and Methanobacteriota_A_1229 after 24 hours relative to baseline samples were higher in CB samples in the PAD group (Fig. 7B). In both CB and GT samples, Patescibacteria demonstrated log fold increase in the non-PAD group but was decreased in the PAD groups (Fig. 7B). Overall, the abundance of genera such as *Enterocloster* (LinDA lfc = 1.37, p = 6.57e^− 08^), *Lachnospira* (LinDA lfc = 1.51, p = 0.0007), *Copromonas* (LinDA lfc = 1.27, p = 0.018), *Lawsonibacter* (LinDA lfc = 0.99, p = 0.009), *Faecalimonas* (LinDA lfc = 0.66, p = 0.0496), and *Clostridium_Q_135822* (LinDA lfc = 1.1, p = 0.02) are higher in GT samples compared to CB samples during RT storage (Fig. 7C). On the other hand, the abundance of genera such as *Streptococcus* (LinDA lfc = −0.4, p = 0.036), *Eggerthella* (LinDA lfc = −0.75, p = 0.02), *Enterococcus_B* (LinDA lfc = −1.91, p = 0.007), and *Rombustia_B* (LinDA lfc = −0.47, p = 0.02) are lower in GT samples during RT storage compared to CB samples (Fig. 7C). We observed specific genera that are associated with reduced abundance in GT media in the PAD group after both 24- and 48-hour storage at RT, including *Clostridium_Q_135822* (24 hours Coeff. = −2.18, p = 0.0003; 48 hours Coeff. = −3.02, p = 0.001), *Eubacterium_R* (24 hours Coeff. = −3.03, p = 4.27e^− 08^; 48 hours Coeff. = −2.2, p = 0.007), *Phascolarctobacterium_A* (24 hours Coeff. = −1.32, p = 0.03; 48 hours Coeff. = −1.54, p = 0.009) and *Ruminococcus_B* (24 hours Coeff. = −2.49, p = 0.0004; 48 hours Coeff. = −2.35, p = 7.64e^− 05^) (Fig. 7D).

We observed a similar pattern of alpha diversity change over time in non-PAD and PAD groups with sharp declines in Shannon and Pielou evenness in CB samples in the PAD group (Fig. 7E). We found significant increases (p < 0.05) in the unweighted UniFrac distance between non-PAD and PAD groups after 24 hours RT storage in both CB and GT samples, followed by significant decline (p < 0.05) at 48 hours (Fig. 7F). Similarly, the weighted UniFrac distance between non-PAD and PAD groups increased (p > 0.0001) after 24 and 48 hours of RT storage compared to baseline in CB samples, whereas it increased (p < 0.05) between 24 and 48 hours in GT samples (Fig. 7F). This suggests that the microbial communities in non-PAD and PAD groups diverge after 24 hours at RT due to differential shifts induced by the CB and GT conditions.

## Discussion

Studies involving the association of human gut microbial biomarkers with various diseases, including cardiovascular diseases are increasingly common. (68, 69) These studies, which typically involve genetic sequencing of the microbiomes from fecal samples, are prone to bias caused by variations in sample collection, processing, and storage. We used culture-independent and culture-dependent methods to demonstrate that storage conditions can differentially affect the microbial communities in the fecal samples of non-PAD and PAD groups.

We found that storage condition caused significant variations in microbial community composition but not in the structure using unweighted UniFrac distance. Unweighted UniFrac distance captures variations in rare species that can be involved in pathogenesis, whereas weighted UniFrac gives more weight to abundant taxa, thereby revealing transient effects of environmental factors on microbial abundance. (70) This makes unweighted UniFrac distance preferable over weighted UniFrac for meta-analyses where samples collected from multiple storage conditions are involved. However, careful consideration is required to validate the group differences observed in different storage conditions, as they show variable within-group dispersion effects (**Supplemental Fig. 2**). All 3 storage conditions revealed similar differences in within-group distances between non-PAD and PAD samples (Fig. 3B), but differences among PAD samples based on change in microbial abundance due to storage conditions was more pronounced (Fig. 2D). However, reduced median distances between non-PAD and PAD groups in CB and GT compared to FR (**Supplemental Fig. 2C and 2D**) make it difficult to determine which group is driving the observed differences in the communities from multiple storage conditions. On the other hand, observed differences in unweighted UniFrac distances within the non-PAD group (Fig. 2C) based on the storage condition may indicate bias caused by subsampling fraction on capturing all rare species. Higher prevalence of rare species in the non-PAD microbiome may be more affected by differences in the storage conditions.

We observed storage condition-associated differences in differential abundance and differential variability between the non-PAD and PAD groups. Differential abundance analysis is commonly performed in cross-cohort microbiome studies to identify biomarkers associated with disease conditions. In both non-PAD and PAD groups, there were fewer (7%) differentially abundant species common to all 3 storage conditions than those that were unique to each storage condition. Differences due to storage conditions in the community abundance pattern (Fig. 2D, **Supplemental Fig. 1 CD, and Supplemental Fig. 2D**) can increase noise and obscure the signal from less abundant biomarkers at lower taxonomic levels. The relative abundance of genera such as *Butyricimonas*, *Copromonas*, *Coprobacter*, and *Parasuturella* is greater in specific storage conditions (Fig. 4E). Furthermore, the higher relative abundance of *Butyricimonas* in the PAD group could result from the nested effect of storage media and RT storage time (Fig. 7D). Since *Butyricimonas* has been implicated in coronary artery disease and serum cholesterol in a mouse fecal microbiota transplantation (FMT) study using fecal samples, (71) and *Parasuturella* from multi-species gastrointestinal isolates has been found to maintain bile acid homeostasis and cholesterol metabolism, (72) which impacts atherosclerosis pathogenesis, our observations highlight the importance of considering sample storage and processing for downstream research applications involving dysbiotic conditions.

Measurements of differential variability can provide valuable insights into the robustness of biomarkers and dysbiosis of taxa in diseased populations. (63) For example, patients with inflammatory bowel disease have increased variability of the gut microbiome and periodic deviations from the healthy plane over time. (73) In our cross-sectional study, we found a higher number of differentially variable species in the PAD group in all 3 storage conditions, which could be attributable to heterogeneity in the PAD phenotype. We also found fewer (16%) differentially variable species that were common to all 3 storage conditions compared to those that were unique to each storage condition. CB samples had 10 differentially variable biomarkers that were also differentially abundant, compared to 7 such biomarkers in FR and GT samples (Fig. 4B; **Supplement Fig. 3B**).

Enrichment of microbial biomarkers in PAD is dependent upon the interaction effect of storage condition and RT storage time. We observed variable effects of RT storage on microbial communities in the non-PAD and PAD groups. For example, *Blautia_A_141780* is more abundant in GT across all samples (Fig. 4C). It also showed enrichment in PAD samples stored in GT for 48 hours compared to non-PAD samples stored in CB at initial storage time (Fig. 4D). Carroll *et al*. (8) also found that the fecal microbiota from patients with irritable bowel syndrome is less stable during RT storage compared to that from healthy controls. Although we did not find an interaction effect of RT storage time with overall community differences between non-PAD and PAD groups, we observed that their unweighted- and weighted UniFrac distances increased with the time of storage (Fig. 7F). This indicates a time-dependent effect of RT storage on community structure, most likely contributed by the Proteobacteria and Firmicutes (Fig. 7A and 7B).

FMT studies using stored frozen samples require the revivification of microbiota. Hence, we compared the revivification potential of GS made from FR and CB samples. Our finding that CFUs were significantly higher from CB than FR samples is in concordance with the findings of Nagata et al. (16) who suggested the use of CB medium when immediate freezing is not available. However, our sequencing data of colonies suggest that higher CFU does not always correspond to a higher number of unique species, since the number of ASVs from CB colonies was fewer compared to those from FR in non-PAD samples (Fig. 5B, Fig. 6A and Fig. 6B). Thus, while cryoprotection of live microbiota with glycerol improves CFU in revivified samples, (74) we found that bacterial diversity is affected by the storage media (Fig. 6C).

We also found that the preservation of observed richness in the gut microbial community from the non-PAD group was greater than the PAD group in all 4 processing conditions (Fig. 6A and 6B). This could partly be due to the altered nutrient composition, higher rate of nutrient depletion, and lack of functional redundancy among microbes in diseased cohort samples. (74) In PAD samples, shared ASVs in colonies from CB samples, regardless of processing condition, were higher (49%) than those from FR samples (39%). This was not observed in the non-PAD samples. This could be due to the selective enrichment of bacteria in the PAD group by the CB media. (16) The majority of human gut microbiota are strict anaerobes that are prone to oxidative stress, and ensuring their survival is essential for FMT studies. Although anaerobic processing of samples stored in CB is ideal for preserving anaerobic gut communities (**Supplemental Fig. 4**), our results suggest the use of immediately frozen samples when anaerobic processing is not feasible (Fig. 6C and 6D).

The strengths of our study are that it addresses a critical gap in understanding the effect of storage conditions on the gut microbiome in diseased states. We used both culture-dependent and culture-independent methods to investigate possible bias that can be introduced by sample storage conditions. We also characterized differences in the revivification of samples after freezing based on initial storage conditions. We compared 2 commonly used storage conditions to immediate freezing, which is the gold standard method. We also prepared samples in each storage condition in parallel immediately after collection. Our findings emphasize the need for adjusting for storage conditions and storage time when they are not uniform across the cohort.

This study has several limitations. First, the use of home fecal sample collection does not allow us to collect and preserve a sample immediately after collection. Immediate return of samples is not feasible for our patient population and all samples were returned within 48 hours, but it is possible that there is some loss in sample integrity during this timeframe. Second, we did not rigorously assess participant handling of samples. Differences in their ability to follow the sample collection protocol could introduce unmeasured variability in the samples. However, no participants reported difficulty following the written and graphical instructions. Third, the modest cohort size does not allow for full characterization of the gut microbiome in PAD, including adjustment for confounding variables. However, the focus of this study was on the effect of storage conditions in PAD, a dysbiotic condition. Fourth, the susceptibility of the dysbiosis to storage conditions may not translate to her disease-associated microbiomes. Finally, we did not perform a metabolomic analysis to understand how storage conditions may impact the functional output of the microbiome, as it was beyond the scope of this study.

In conclusion, we demonstrate that home fecal sample collection is feasible among patients with PAD; that fecal sample storage conditions have differential effects on the gut microbiome in donors with PAD, a condition known to be associated with dysbiosis; and that revivification of samples from dysbiotic cohorts for downstream FMT requires consideration of sample storage media and processing methods.

We suggest that future studies ensure uniform storage and processing methods for improved microbiome-based biomarker discovery, that cross-cohort studies using non-uniform storage methods adjust for these important covariates, and that characterizations of dysbiosis account for potential differences related to the storage condition.

## Supplementary Files

This is a list of supplementary files associated with this preprint. Click to download.

- Supplementarymaterial.docx

## Figures and Tables

**Table 1.** Demographic and clinical variables.

Variable*	Non-PAD n=14	PAD n=21
Age (years)		
Mean ± SD	40.93 ± 15.55	70.14 ± 9.26
Range	23–69	54–90
Female sex	5 (35.71)	10 (47.62)
Race		
Caucasian	7 (50.00)	10 (47.62)
Black	2 (14.29)	9 (42.86)
Other	5 (35.71)	2 (9.52)
Body mass index (kg/m^2^)		
Mean ± SD	28.53±4.53	27.56±3.73
Diabetes mellitus	0	15 (71.43)
Smoking (current or former)	7 (50.00)	17 (80.95)
Statin medication	2 (14.29)	20 (95.24)

* Data presented as n (%) unless otherwise indicated.

SD, standard deviation

## Data Availability

The datasets analyzed during the current study are available in the National Center for Biotechnology Information’s Sequence Read Archive under the BioProject accession number PRJNA1309752.
